# Determination of antimicrobial susceptibility and biofilm production in *Staphylococcus aureus* isolated from white coats of health university students

**DOI:** 10.1186/s12941-019-0337-6

**Published:** 2019-11-28

**Authors:** Isabela Rotta Batista, Amanda Caroline Lima Prates, Bruna de Souza Santos, Josimara Cristina Carvalho Araújo, Yan Christian de Oliveira Bonfim, Marcus Vinícius Pimenta Rodrigues, Glilciane Morceli, Jossimara Polettini, Andressa Cortes Cavalleri, Lizziane Kretli Winkelstroter, Valéria Cataneli Pereira

**Affiliations:** Universidade do Oeste Paulista - UNOESTE, Rua José Bongiovani, 700 - Cidade Universitária, Presidente Prudente, SP CEP 19050-920 Brazil

**Keywords:** *S. aureus*, MRSA, Biofilm, White coats

## Abstract

This study aimed at detecting *Staphylococcus aureus* from white coats of college students and characterizing antimicrobial susceptibility and biofilm production. Bacterial samples (n = 300) were obtained from white coats of 100 college students from August 2015 to March 2017 *S. aureus* was isolated and it´s resistance profile was assessed by antimicrobial disk-diffusion technique, screening for methicillin-resistant *Staphylococcus aureus* (MRSA), detection of *mecA* gene by PCR, and determination of staphylococcal cassette chromosome *mec* (SCC*mec*) by multiplex PCR. Congo red agar (CRA) and *icaA* and *icaD* genes by PCR were used for biofilm characterization. *S. aureus* was identified in 45.0% of samples. Resistance of *S. aureus* sample to antimicrobial was seen for penicillin (72.59%), erythromycin (51.85%), cefoxitin (20.74%), oxacillin (17.04%), clindamycin (14.81%) and levofloxacin (5.18%). MRSA was detected in 53.3% of the samples with SCC*mec* I (52.8%), SCC*mec* III (25%) and SCC*mec* IV (11.1%). Biofilm production was observed in 94.0% *S. aureus* samples. These data show that biosafety measures need to be enhanced in order to prevent dissemination of multiresistant and highly adhesive bacteria across other university settings, relatives, and close persons.

## Introduction

Personal protective equipment (PPE), such as white coats, is used as biosafety measure, mainly recommended to healthcare professionals working in unwholesome environments with variable risk. [1]. Commensal microorganisms, most commonly *Staphylococcus aureus*, can be easily disseminated through white coats [2]. This bacteria is commonly found in nasal mucosa and can become pathogenic, triggering infections such as skin boils and pimples, cellulitis, bacteremia, pneumonia, osteomyelitis, and acute endocarditis, among others [3].

Inappropriate white coat use is an important way for transmission of methicillin-resistant *S. aureus* (MRSA) [4]. MRSA infections are difficult to treat due to it´s resistance to all beta lactam antimicrobials, leaving few alternatives for treatment [5].

The assessment of *S. aureus* susceptibility to methicillin is key to proper infections treatments without unnecessary use of vancomycin, since this agent can lead to several therapeutic complications, in spite of being a first-line and less expensive antimicrobial for MRSA infections [6]. Although vancomycin has been used since 1958, hospital samples have already show reduced susceptibility of *S. aureus* to vancomycin (vancomycin-intermediate *Staphylococcus aureus*-VISA) [7].

In addition to antimicrobial resistance, *S. aureus* has the ability to produce a complex molecular structure named biofilm in both biotic and abiotic surfaces, such as white coats. Biofilm promotes adhesion to substrate and protects the microorganism from host immune response and antimicrobial action through an extracellular matrix composed by proteins, carbohydrates and deoxyribonucleic acid (DNA) [8]. Biofilm can thus be associated to bacteria cells adhesion to the fabric white coats of professionals who use that PPE.

College students frequently wear white coats as mandatory PPE during pedagogical activities in hospitals and laboratories settings. Moreover, some students also wear coats in improper settings like libraries, cafeteria, and even during their journey back home [1]. According to Muhadi et al. [9], it is very common to find white coats laying down on chairs and outside laboratory and hospital settings, potentially causing pathogen dissemination.

Considering that multiresistant bacteria can be carried across different settings, it is especially important to evaluate the susceptibility of *S. aureus* from health students’ lab coats to antimicrobials when they wear such PPE item in different settings while attending classes. The characterization of *S. aureus* isolated from lab coats can help preventing these microorganisms from spreading through different university settings. This is why this study aimed at detecting *S. aureus* from white coats worn by college students and characterizing this microorganism in terms of antimicrobial susceptibility and biofilm production.

## Methods

The present study is a cross-sectional trial, registered and approved by the Universidade do Oeste Paulista Committee for Ethics in Research, Presidente Prudente, Brazil. Participants were Biomedical Sciences students from the third semester on, i.e. when they started clinical practice. Participants answered a questionnaire regarding personal information related to white coat use: semester of the course, gender, laboratories they attended during the period of study, coat cleaning frequency and purpose of this PPE use.

Bacterial samples were obtained from white coats of college students from August 2015 to March 2017. Samples were collected by rolling a sterile swab moistened with sterile saline solution (0.85%) through three areas: collar, pocket and sleeve. Immediately upon collection, samples were forwarded to the Universidade do Oeste Paulista Microbiology Laboratory, Presidente Prudente, Brazil, where they were kept for 24 h at 37 °C in brain–heart infusion (BHI) broth for growth and subsequently seeded in mannitol salt agar for selection of *Staphylococcus* samples. After growth on agar plates, colony samples were submitted to GRAM staining and catalase and coagulase test for differentiation of *S. aureus* and coagulase-negative staphylococci (CoNS). Samples obtained were frozen at − 70 °C in nutrient broth with 10% glycerol.

Antimicrobial sensitivity was tested by drug diffusion in agar using disks infused with oxacillin, cefoxitin, penicillin, clindamycin, erythromycin, and levofloxacin according to Clinical Laboratory Standards Institute criteria [10].

Screening method with oxacillin (6 μg/ml) and NaCl (4%)-enriched Mueller–Hinton Agar was used to identify MRSA. Inoculums were standardized according to Pereira et al. [11] and MRSA detection was assessed by growth of at least one colony on the agar surface.

*Staphylococcus aureus* were seeded in Congo red agar (CRA); following incubation at 37 °C for 24–48 h, biofilm-producing colonies stained in black, whereas isolated staining in red to burgundy were considered non producers [12].

Bacterial DNA extraction was performed by using the Illustra tissue and cells prep genomic mini spin kit (GE Healthcare, Little Chalfont, UK), according to manufacturer´s instructions, following an adapted protocol described by Pereira et al. [13]. DNA quality and concentration were measured by assessing the 260/280 nm absorbance ratio (Gen5, Epoch, Bio Tek, Winooski, VT). The obtained DNA was stored at − 20 °C up to processing.

Detection of *mecA* gene was performed by conventional PCR reactions according to the protocol described by Murakami et al. [14]. Strains of *S. aureus* ATCC 33591 (positive control) and ATCC 25923 (negative control) were included in all reactions. Staphylococcal Cassette Chromosome *mec* (SCC*mec)* typing in MRSA strains was performed by *multiplex* PCR as described by Oliveira et al. [15]. PCR reactions for detection of biofilm formation-related genes (*icaA* e *icaD*) were performed according to Arciola et al. [16]. Effectiveness of all amplified reactions was monitored by electrophoresis in 2% agarose gel stained with ethidium bromide.

Statistical analyses were made through R program (3.3.2 version) and it‘s statistical package. Fisher’s exact test (table 2 × 2) was used to assess antimicrobial resistance and susceptibility. Sensitivity and specificity were evaluated by comparing the phenotypic methods used and PCR for the detection of MRSA and genes involved in biofilm synthesis [17]. Agreement between phenotypic and genotypic tests was evaluated using the kappa index [18].

## Results

A total of 300 samples from sleeve, pocket, and collar of 100 white coats were included in this study. *S. aureus* was detected in 135 samples (45%), whereas coagulase-negative staphylococci, bacilli, and unidentified microorganisms were found in 20, 69 and 76 samples, respectively. *S. aureus* detection rate was significantly higher (p < 0.001). *S. aureus* was most often isolated from collar (n = 53, 39%), followed by sleeve (n = 45, 33%) and pocket samples (n = 37, 28%).

Table 1 shows the results of disk-diffusion assessment of microbial resistance for 135 *S. aureus* isolates. MRSA was identified by screening test on 44 samples (32.6%) as shown Table 2.

**Table 1:** Antimicrobial susceptibility in *S. aureus* by disk-diffusion technique

Antimicrobial	Resistant
	N (%)
Pen	21 (15.5)
Ery	3 (2.22)
Cef/Lev	1 (0.74)
Pen/Clin	3 (2.22)
Pen/Cef	1 (0.74)
Pen/Ery	34 (25.18)
Ery/Oxa/Clin	1 (0.74)
Pen/Oxa/Cef	5 (3.7)
Pen/Cef/Lev	1 (0.74)
Pen/Ery/Oxa	4 (2.96)
Pen/Ery/Cef	8 (5.92)
Pen/Ery/Clin	7 (5.18)
Pen/Oxa/Cef/Clin	1 (0.74)
Pen/Ery/Oxa/Cef	3 (2.22)
Pen/Ery/Oxa/Clin	1 (0.74)
Pen/Ery/Oxa/Cef/Clin	3 (2.22)
Pen/Ery/Oxa/Cef/Lev	3 (2.22)
Pen/Ery/Cef/Clin/Lev	1 (0.74)
Pen/Ery/Oxa/Clin/Lev	1 (0.74)
Pen/Ery/Oxa/Cef/Clin/Lev	1 (0.74)
Sensitive^a^	32 (23.70)

*Pen* penicillin, *Ery* erythromycin, *Oxa* oxacillin, *Cef* cefoxitin, *Clin* clindamycin, *Lev* levofloxacin, *N* number of *S. aureus* samples

^a^*S. aureus* sensitive to all antimicrobials

**Table 2:** Sensibility and specificity to oxacillin in *S. aureus* samples by phenotypic and genotypic methods

Phenotypic methods	*mecA*		Sensibility	Specificity	Kappa coefficient
	Positive	Negative			
	N = 72	N = 63	%	%	*k* (interpretation)
Oxacillin (10 µg)	13	10	18.0	84.1	0.02 Non-concordance
Cefoxitin (30 µg)	17	11	23.6	82.5	0.07 Non-concordance
Screening^a^	26	18	36.1	82.5	0.35 Satisfactory concordance

N: number of *S. aureus*; *k*: Kappa coefficient

^a^Oxacillin (6 μg/ml) and NaCl (4%)-enriched Mueller–Hinton Agar

**Table 3:** Determination of antimicrobial resistance by MRSA, according to *SCCmec* types

Antimicrobial	N	SCC*mec*			
		Type I (%)	Type III (%)	Type IV (%)	Untyped (%)
Erythromycin	38	50	30	8	12
Clindamycin	10	50	40	10	0
Penicillin	50	54	26	8	12
Levofloxacin	3	100	0	0	0

All isolates were examined for *mecA* gene detection in order to confirm MRSA phenotypic results from screening and disk-diffusion with oxacillin and cefoxitin. Positivity for *mecA* gene was observed in 72 *S. aureus* samples (53.3%). Sensitivity and specificity results from phenotypic tests in comparison with *mecA* gene and Kappa’s concordance analysis are shown in Table 2.

Isolates positive for *mecA* gene were examined by *multiplex* PCR technique for SCC*mec* typing. Thirty-eight (52.8%) *S. aureus* samples presented SCC*mec* I, 18 (25.0%) SCC*mec* III, and 8 (11.1%) SCC*mec* IV. Typing was not possible in eight (11.1%) *S. aureus* samples. Table 3 shows susceptibility of these strains to other antimicrobials tested.

Of all identified *S. aureus,* 127 (94.0%) were biofilm producers as shown by black colonies on Cong red agar surface. Of all biofilm-producing isolates, 42 (33.0%) were MRSA.

Results regarding biofilm formation-related genes showed 109 (80.7%) strains positive for *icaA* gene and 127 (94.0%) for *icaD* gene. In 105 samples (77.8%) both genes were concomitantly detected; and 99 samples (73.3%) were shown to be positive on CRA. Sensibility and specificity of CRA regarding *icaA* was 94% and 7.7%, respectively. On the other hand, sensitivity was 94% and specificity 0% for *icaD*. There was minimum agreement between CRA and *icaA* gene (k = 0.03), and no agreement whatsoever between CRA and *icaD* gene (k = − 0.06).

Data on inside and outside university white coat use showed that *S. aureus* was isolated in 64 (77.1%) white coat samples from female participants (n = 83) and in 13 (76.5%) white coat samples from male participants (n = 17).

**Fig. 1:**
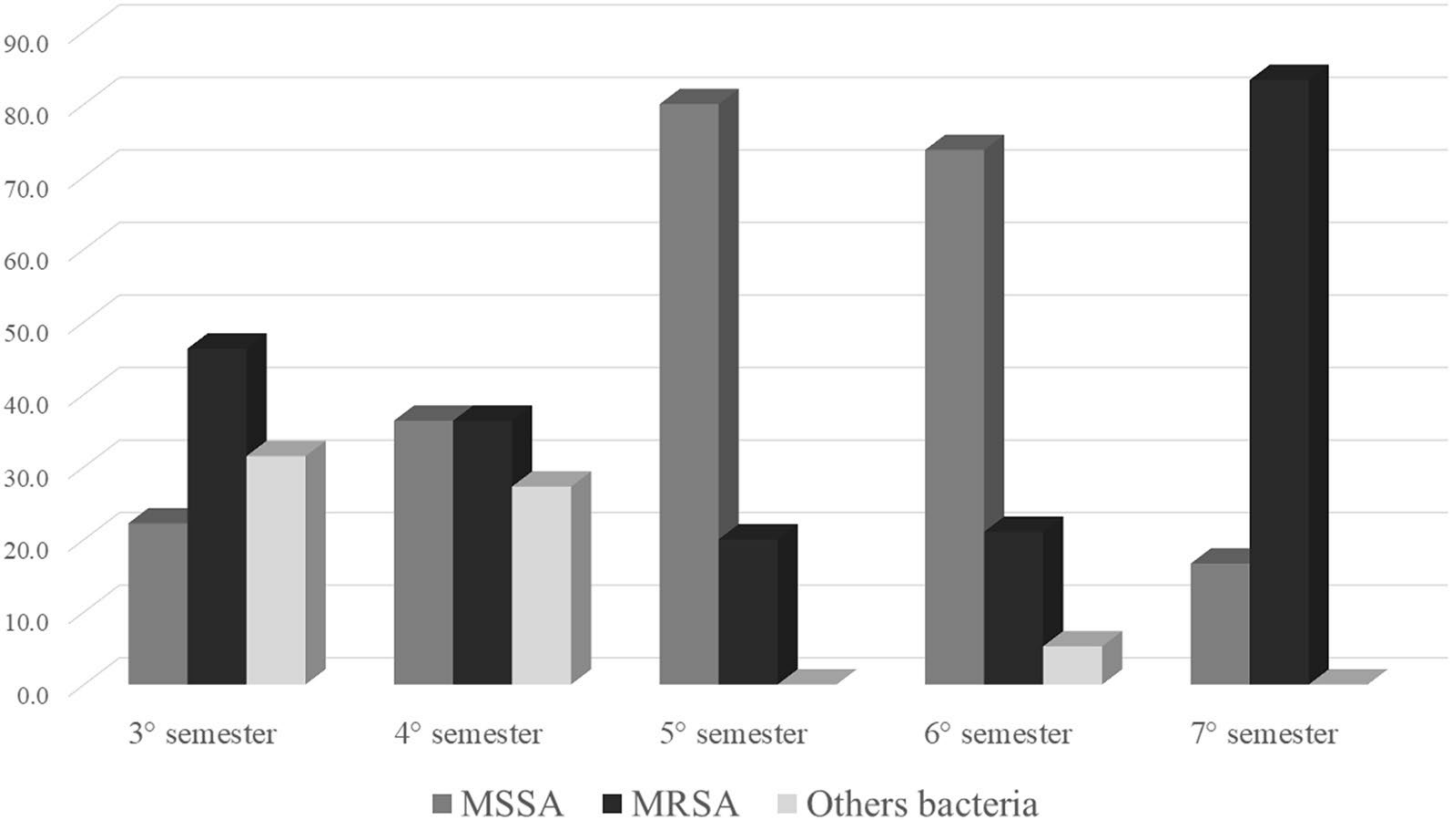
Colonization frequency of white coats by *S. aureus* and MRSA according to the different current course period of the participants

Colonization frequency by Methicillin-sensitive *S. aureus* (MSSA) and MRSA according to the different current course periods of participants is shown in Fig. 1. Highest rates of all three microorganisms are seen in samples from 3rd semester students.

**Fig. 2:**
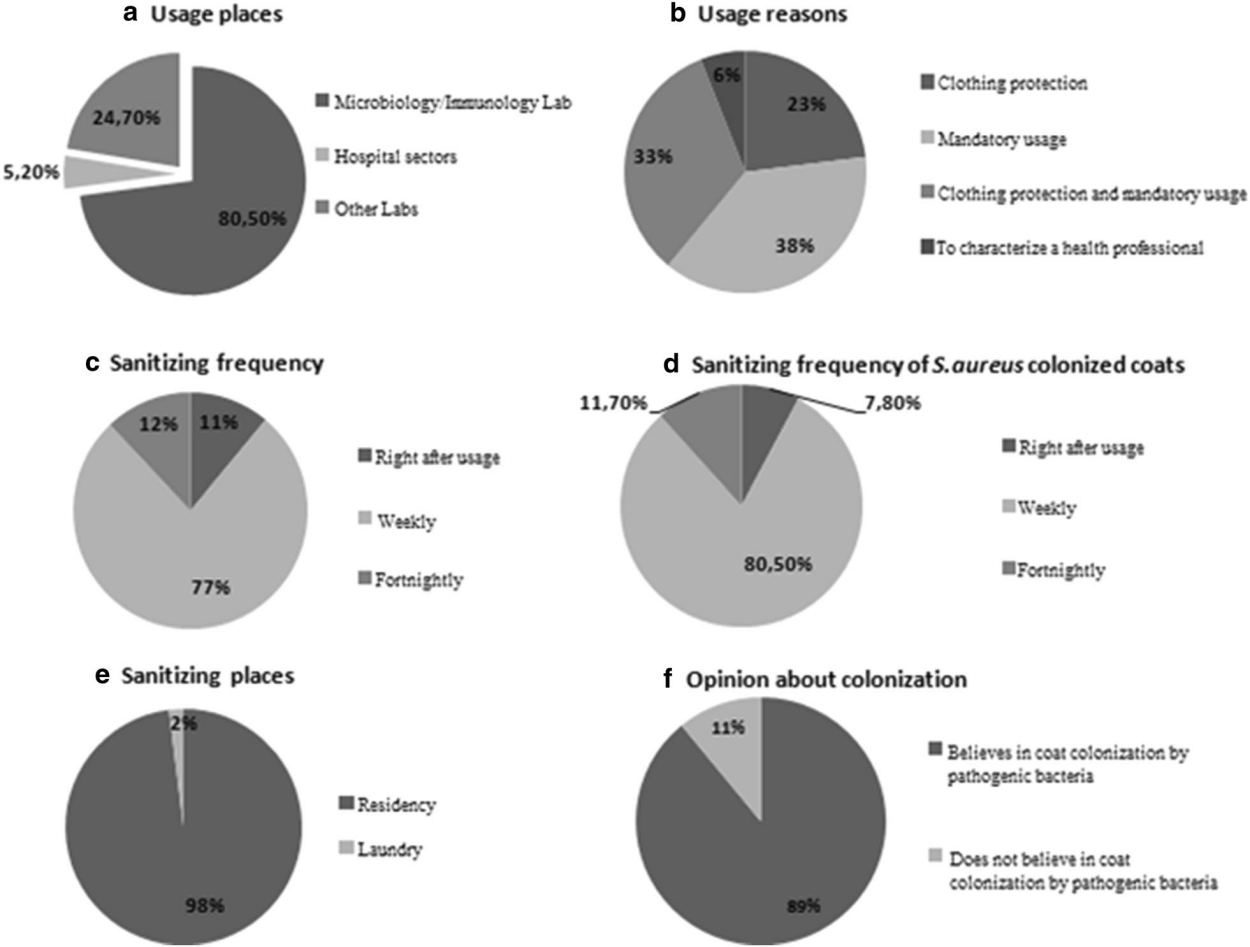
Questions on coat usage by students of the Biomedicine course. **a** Usage places. **b** Usage reasons. **c** Sanitizing frequency. **d** Sanitizing frequency of *S. aureus* colonized coats. **e** Sanitizing places. **f** Opinion about colonization

Figure 2 shows results regarding coat usage. Most students stated that wearing a white coat aims at protecting clothes and is a mandatory PPE item at Microbiology and Immunology lab, settings where white coats are mostly worn by students. Moreover, 98% of the students perform sanitization procedures at home, and about 80% do it weekly, regardless of *S. aureus* colonization (Fig. 2). Interesting, almost 90% of the students are aware that this PPE item is potentially colonized by bacterial pathogens.

## Discussion

White coats are PPE items widely used by health professionals and students during their academic education. According to Brazilian law (n°. 14.446), the use of this item outside workplace is strictly forbidden, violators being subject to severe financial penalties. Wearing a white coat in improper places can lead to risk of pathogen transmission. This study identified a 45.0% contamination rate of college students´ white coats with *S. aureus*, a pathogen playing a major role in community-acquired infections.

The rate of *S. aureus* isolates found herein is higher than in other studies. Fenalte et al. [19] have detected this microorganism in 19.8% of samples from coats worn by nursing professionals in a midsize hospital, while Margarido et al. [20] identified *S. aureus* in 31.6% of samples from nursing students. The data presented in this study highlight *S. aureus* colonization in coats worn by students less exposed to hospital environment than those in other health science courses.

Since genotypic techniques are considered gold standard for MRSA detection, the high rate of such strains found in students´ coats is a major concern, as other studies have shown lower rates in white coat samples from health professionals with higher exposure to hospital environment. Treakle et al. [21] have detected 23% of *S. aureus* isolated from 149 samples from coats worn by health professionals, and 6 were identified as MRSA. Additionally, Fenalte et al. [19] described a 4.7% rate of MRSA in isolates from coats worn by nursing technicians. Low sensitivity and specificity of phenotypic methods in the present study reflect the difficulty in detecting MRSA, thus highlighting the importance of using molecular methods to detect these strains.

Another alarming result from the present work refers to SCC*mec* typing in MRSA, which showed high rates of types I and III, the most prevalent types in hospitals settings. A study with 62 MRSA isolated from six healthy professionals and 56 patients has shown SCC*mec* III as the most frequent type (76%), followed by SCC*mec* IV (11.2%), SCC*mec* I (4.8%) and SCC*mec* V (3.2%). In this same study, the authors point out that SCC*mec* I was isolated in blood culture only, whereas the SCC*mec* IV and V were associated to wounds and urine samples and SCC*mec* III was isolated from all analyzed clinical materials [22].

More than 50% of MRSA isolates with SCC*mec* I showed resistance to erythromycin, clindamycin, penicillin, and levofloxacin. Resistance to erythromycin, clindamycin and penicillin was also observed in MRSA with SCC*mec* III and IV. One of the main mechanisms of acquiring genes related to antimicrobial resistance involves acquisition of mobile genetic elements, mainly among larger SCC*mec.* Deletion events are also very common as a mechanism of acquiring genes related to antimicrobial resistance, as a MRSA strain can be self-modified to MSSA or lose antimicrobial resistance genes [22].

Ninety-four percent of *S. aureus* detected were biofilm producers, indicating that production of this polysaccharide is an important mechanism providing closer adhesion to coat fabrics. Adhesion to coat surface can be related to the polysaccharide intercellular adhesin (PIA), mediated by *icaA* and *icaD* genes and different mechanisms. According to Fredheim et al. [23], extreme conditions such as heat, pressure, and environmental chemical can influence biofilm formation. Furthermore, certain *Staphylococcus* species can develop alternatives mechanisms, such as PIA-independent biofilm formation.

Congo Red Agar test showed a 94% sensitivity rate for *icaA* and *icaD* genes. Although CRA method has shown low specificity compared to *icaA* and *icaD* genes, as well minimal agreement with *icaA* gene and no agreement with *icaD* gene, these results reflect the importance of studying other biofilm production mechanisms, since there are other genes, like *bap*, encoding biofilm-associated protein [24].

Although 87% of students with *S. aureus*-colonized coats are aware of the potential of this PPE item to carry bacterial pathogens, it seems, in view of the high colonization rates on white coats herein detected, that biosafety measures need to be reinforced. As for coat sanitization, 80.5% of coats colonized by *S. aureus* are sanitized weekly and 97.4% of the students reported performing the procedure at home. Silva et al. [25] point out that health professionals should be trained for better knowledge of measures involving precaution, proper use, and adequate maintenance of their white coats.

Students should therefore receive proper education regarding biosafety procedures, especially after classes and lab research activities. Ideally, white coats should be disposable, but since this practical is not viable it is mandatory that their use follow rigorous sanitization measures, including separate laundering with powder detergent followed by drying and ironing, which can prevent the possibility of bacterial pathogen dissemination, according to Margarido et al. [20]. These authors collected samples from white coats sanitized according to standard procedures, i.e. laundering with powder detergent, drying, and ironing, and could demonstrate absence of bacterial growth in 100% of samples. The present study shows that biosafety measures need to be enhanced in order to prevent dissemination of multiresistant and highly adhesive bacteria across other university settings, relatives, and close persons.

## Data Availability

The datasets used and/or analysed during the current study are available from the corresponding author on reasonable request.

## Abbreviations

PPE: personal protective equipment
MRSA: methicillin-resistant *S. aureus*
MSSA: methicillin-sensitive *S. aureus*
VISA: vancomycin-intermediate *Staphylococcus aureus*
BHI: brain heart infusion
CoNS: coagulase-negative staphylococci
CRA: Congo Red Agar
SCC*mec*: staphylococcal cassette chromosome *mec*
